# CHAT SA: Modification of a Public Engagement Tool for Priority Setting for a South African Rural Context

**DOI:** 10.34172/ijhpm.2020.110

**Published:** 2020-07-08

**Authors:** Aviva Tugendhaft, Marion Danis, Nicola Christofides, Kathleen Kahn, Agnes Erzse, Marthe Gold, Rhian Twine, Audrey Khosa, Karen Hofman

**Affiliations:** ^1^SAMRC/Wits Centre for Health Economics and Decision Science - PRICELESS, School of Public Health, Faculty of Health Sciences, University of Witwatersrand, Johannesburg, South Africa.; ^2^Department of Bioethics, National Institutes of Health, Bethesda, MD, USA.; ^3^School of Public Health, Faculty of Health Sciences, University of the Witwatersrand, Johannesburg, South Africa.; ^4^MRC/Wits Rural Public Health and Health Transitions Research Unit - Agincourt, School of Public Health, Faculty of Health Sciences, University of the Witwatersrand, Johannesburg, South Africa.; ^5^New York Academy of Medicine, New York City, NY, USA.

**Keywords:** Priority Setting, Public Engagement, Rural Health, South Africa

## Abstract

**Background:** Globally, as countries move towards universal health coverage (UHC), public participation in decisionmaking is particularly valuable to inform difficult decisions about priority setting and resource allocation. In South Africa (SA), which is moving towards UHC, public participation in decision-making is entrenched in policy documents yet practical applications are lacking. Engagement methods that are deliberative could be useful in ensuring the public participates in the priority setting process that is evidence-based, ethical, legitimate, sustainable and inclusive. Methods modified for the country context may be more relevant and effective. To prepare for such a deliberative process in SA, we aimed to modify a specific deliberative engagement tool – the CHAT (Choosing All Together) tool for use in a rural setting.

**Methods:** Desktop review of published literature and policy documents, as well as 3 focus groups and modified Delphi method were conducted to identify health topics/issues and related interventions appropriate for a rural setting in SA. Our approach involved a high degree of community and policy-maker/expert participation. Qualitative data were analysed thematically. Cost information was drawn from various national sources and an existing actuarial model used in previous CHAT exercises was employed to create the board.

**Results:** Based on the outcomes, 7 health topics/issues and related interventions specific for a rural context were identified and costed for inclusion. These include maternal, new-born and reproductive health; child health; woman and child abuse; HIV/AIDS and tuberculosis (TB); lifestyle diseases; access; and malaria. There were variations in priorities between the 3 stakeholder groups, with community-based groups emphasizing issues of access. Violence against women and children and malaria were considered important in the rural context.

**Conclusion:** The CHAT SA board reflects health topics/issues specific for a rural setting in SA and demonstrates some of the context-specific coverage decisions that will need to be made. Methodologies that include participatory principles are useful for the modification of engagement tools like CHAT and can be applied in different country contexts in order to ensure these tools are relevant and acceptable. This could in turn impact the success of the implementation, ultimately ensuring more effective priority setting approaches.

## Background

**Key Messages**

** Implications for policy makers**

- Policy-makers may benefit from the inclusion of the public in determining the priority setting agenda, not simply from the inclusion of the public once the potential options have been predefined.
- Modification of public engagement tools is resource and time intensive and should take place ahead of time to ensure the tool is available for use during priority setting processes.
- The modified CHAT (Choosing All Together) tool can serve as a basis for further modifications for different settings in South Africa and elsewhere in Africa.
- Participatory methods are feasible in the modification of a public engagement tool like CHAT and can be applied in different country contexts in order to ensure these tools are relevant and acceptable thereby strengthening the priority setting process.

** Implications for the public**

Cost effectiveness analyses are becoming increasingly important in allocation decisions for health coverage, but this process alone does not comprehensively consider public values, and public deliberation is therefore a potential mechanism to fill this gap by incorporating social values into the priority setting process. Public engagement on issues of decision-making and prioritization could be key to the success of setting priorities and context-specific tools to facilitate this process could be useful. Community engagement is important in not only identifying social values for final coverage options for health service packages but also for identifying the initial topics/ issues and specific interventions that should be weighed up when thinking about these packages. By bringing the voices of decision-makers and the public together the priority setting agenda can be set not just by experts but also by the public. This could ultimately ensure priority setting approaches that are not only evidence-based, but ethical, legitimate, sustainable and inclusive.

Public engagement in priority setting for health is the practice of actively involving members of the public in the decision-making activities of health policy development, which can also include health service design and planning.^1,2^ The moral imperative of transparency and public engagement is widely recognized by health experts and ethicists.^3-6^ These perspectives demonstrate that health priority-setting should reflect the values of the public, and the decision-making process, therefore, should include their involvement. Beyond improving the ability of the public to influence decisions on issues that affect their lives, public engagement has the potential to reinforce the legitimacy and the public acceptability of the decision-making process and its outcomes; increase the success rate of policy implementation; manage community expectations and improve public understanding of the issues considered.^7,8^ Public engagement in decision-making is also viewed as particularly valuable to inform difficult decisions about priority setting and resource allocation as countries move towards universal health coverage (UHC).^9,10^

In low- and middle-income countries, public engagement for priority setting for health, particularly at the local level, is promoted. There are some examples such as those in Uganda, Tanzania, Indonesia, and India where public engagement structures have been put in place and where there has been some degree of participation and subsequent impact, albeit small scale, on decision-making.^2,11,12^ The effectiveness of public engagement in decision-making in these settings, however, is unclear and in many circumstances, even where the public has a constitutional right to be involved, the processes for engagement do not result in meaningful participation.^2,11,12^ This is due in part to political, practical and cultural factors including barriers to physical access, poverty, social exclusion, disconnect between local and national priorities, time constraints and lack of oversight among others.^2^

While public engagement is widely endorsed, many questions remain about how best to achieve this.^2,13-15^ Methods of engaging the public in priority setting fall broadly into non-deliberative and deliberative processes. Non-deliberative methods may be consultative in nature, but they do not provide the same degree of consideration, nor of two-way communication and debate that deliberative methods offer. Deliberative methods involve deeper engagement and considerations of choices among a selected group of individuals.^14^ According to the American Institute for Research:

“*Public deliberation is a unique way of convening a diverse group of citizens to consider an ethical or values-based dilemma and then weigh alternative — often competing — views…[it] rests on the democratic principle that important societal decisions are best made by policy-makers in partnership with an informed public.”*^16^

Abelson and colleagues outline 3 components of public deliberation which enhance data richness on public attitudes and values and enable participants to develop ideas and priorities through interaction. These include: providing participants with factual and balanced information that provides a shared knowledge base, ensuring that individuals with diverse perspectives are represented, creating a setting where values and moral claims/opinions can be voiced and challenged.^14^

Deliberative engagement processes have been proposed for priority setting where issues are complex and there are diverse public perspectives. This type of engagement is considered useful in identifying and balancing individual and societal values, and concerns and driving collective outputs.^17^ There are a number of deliberation methods that range in structure, number of participants, duration of engagement, number of sessions, and extent of educational materials.^14,17^

One method of public deliberation is through the use of the CHAT (Choosing All Together) tool^[1]^. CHAT is a game-like exercise where participants work individually and then in groups to distribute a limited number of stickers on a board as they select from a wide range of options. The stickers, which represent the available budget, are only able to cover approximately 60% of the options on the board. CHAT simulates priority setting processes whereby limited resources are available for a wide variety of interventions, and trade-offs are inevitable.^18^ CHAT was designed to overcome some of the barriers of public participation through facilitating a deliberative and interactive process that encourages group decision-making.^18,19^ It was originally developed in 2000 by researchers at the University of Michigan and the US National Institutes of Health to include the public in creating health insurance packages and has since been used and modified to explore coverage trade-offs within a variety of audiences, often low-income groups, and in relation to placing priorities on various types of assets. This has included, among others, engaging community members in California to define a basic health coverage package, engaging low-income employees in the United States to identify employee benefits packages, including low-income urban residents in identifying priorities to address the socio-economic determinants of health, involving patients in developing a coverage package for advanced cancer care and for mental health, engaging members of the public in Switzerland to identify a health insurance package and engaging low-income rural residents in India in developing a micro-insurance package.^20-27^ Beyond health related issues CHAT has been used more broadly for other types of priority setting, for example identifying priorities for research.^25^

The application of CHAT in India demonstrates that the tool can be useful for low- and middle-income countries and for rural communities. The need for the tool in the Indian rural setting arose from the gap between benefit packages available and those reflecting the priorities of the poor. The outcome of the exercise demonstrated that CHAT improved awareness of resource allocation and trade-offs among participants and, in this particular setting, enhanced both willingness to join health insurance and willingness to pay for it.^23^

CHAT has been translated into various languages, and has been adapted for computer and web-based use.^18,28,29^ Because CHAT includes various rounds whereby participants first work on their own to prioritize as individuals and then in a group to make decisions together, the exercises have at times resulted in decisions being made that are not based only on personal preferences but on societal priorities and values.

In South Africa (SA), where deliberative democratic principles prevail and the right to health is protected in The Bill of Rights, public engagement in priority setting for health is entrenched in various policy documents and formalized in the National Health Act, which makes provision for the establishment of community health committees, hospital boards and local health councils.^30^ While the Act stipulates that provincial departments of health must develop legislation which identifies the specific functions of the health committees, the intention is that the members of these committees should ensure community participation in the governance of and priority setting process for local clinics.^31^ While the political climate is in theory supportive of public engagement in priority setting for health at various levels, and while some Community Health Committees do exist with public representation, the role of these bodies is poorly defined, they do not function optimally and members have little input in decision-making.^32^ At the national level, beyond public commenting and parliamentary consultations no formal structure for more meaningful public engagement in priority setting exists.

SA is committed to delivering quality UHC over the next few years through a National Health Insurance (NHI) funding scheme. Policy-makers, just like those in other countries moving towards UHC, are facing challenges in terms of what and who to cover with a limited budget, and local level decision-makers may face service delivery dilemmas. Priorities will need to be set that reflect health needs, economic resources, professional and societal values, and political considerations, among others.^33^ The South African National Health Insurance Bill identifies cost effectiveness standards as key components for determining and refining the interventions that will be covered by NHI.^34^ While cost effectiveness analyses are essential in guiding decision-making, particularly for resource allocation, this process alone does not comprehensively consider social values. Public engagement on issues of decision-making and prioritization could be key to the success of setting priorities and context-specific tools to facilitate this process could be useful. Deliberative engagement methods could be useful in this regard because many of the impending coverage decisions in SA are complex and will require identifying and balancing individual and societal values and concerns. Context specific tools provide a potential mechanism to incorporate social values into the priority setting process in a meaningful way. The approach for developing a context specific tool is important so that the method for engagement is acceptable and reflects considerations that are important to the public and not simply pre-defined by the policy-makers. These considerations should incorporate broad topics/issues as well as specific interventions to address them. A participatory approach for developing/modifying a deliberative engagement tool involving input of community members from the start could be effective in ensuring inclusive priority setting approaches that are evidence-based, ethical, legitimate, sustainable and acceptable.

To prepare for such a deliberative process in SA, we aimed to modify the CHAT tool for a rural community context. We selected CHAT as the deliberative engagement tool because it simulates priority setting processes and has generated positive results in its ability to engage different audiences around resource allocation decisions, particularly amongst other low-income populations and in multiple cultural settings.^23^ Our specific aims were to (*a*) identify health topics/issues for the rural context and specific interventions related to these topics/issues; (*b*) estimate the cost of the specific interventions; (*c*) convert intervention costs into sticker amounts for the CHAT game board to depict the monetary value of the interventions; (*d*) develop context specific scenario cards to demonstrate consequences of choosing interventions during the CHAT exercise, and (*e*) translate the materials into the local language.

 Other adaptations of CHAT have followed similar steps but many have not included the same degree of community and policy-maker participation as our modification methodology. They have also not been documented in detail. In this paper we present all stages of the modification process, using an iterative participatory approach to adapting the CHAT tool. It is the first time CHAT has been modified for the South African context.

## Methods

###  Study Site 

The CHAT tool was modified for use in the Agincourt Health and Socio-Demographic Surveillance System (HDSS) study area (https://www.agincourt.co.za/), located in Bushbuckridge municipality in Mpumalanga province. The site, typical of rural areas in SA, is comprised of 31 villages, 20 000 households and a population of approximately 111 500.^35,36^ There are 2 health centers and 6 satellite clinics in the site and 3 district hospitals within 20-60 km. Pipe-borne water is not available to most households and sanitation systems are poor. Electricity is available in all villages, but is unaffordable for most and few tarred roads exist. Every village has at least one primary school and most have a high school but the quality of education is poor^37^ and unemployment rates are high with labour-related out-migration commonly occurring. Life expectancy at birth is 61 for males and 70 for females,^35^ with significant socioeconomic disparities across different indicators.^38^

###  Adaptation of the Choosing All Together Tool - Data Collection 

 In order to modify CHAT for use in Agincourt HDSS study area we followed a 5-step approach:

####  1. Rapid Desktop Review

First a rapid review^39^ of national health policy documents was conducted to identify national health topics/issues and related interventions that were a priority for the country. The starting points were the most recent national health policy documents in 2017: the transcript of the 2017 SA treasury budget speech where health spending was mentioned, and the NHI White Paper of 2015.^40^ We then selected other national health documents that included any of the health topics/issues identified in the budget speech or the NHI White paper. Eleven documents were included. Finally, we included provincial and district level policy to identify any health topics/issues and related interventions specific to the Bushbuckridge rural context which had not been identified from the national documents. Three additional documents were included. The documents that were included in the review are shown in Supplementary file 1. The documents were scanned in their entirety and any specific interventions related to the health topics/issues were identified and captured in a Microsoft Excel sheet.

####  2. Focus Group Discussions

 Next, we conducted a focus group discussion (FGD) with each of the following groups: home-based care (HBC) service providers in the Agincourt HDSS study area, provincial and district level experts, and national policy-makers, in order to identify which health topics/issues and related interventions (solutions) each group thought were important. The health topics/issues related to categories of disease (eg, HIV/AID and tuberculosis [TB]) or focus area ( eg, mental health) or broader issues like “access,” while the interventions (solutions) related to specific activities and services that would address the health topics/issues, for example, the provision of contraceptives at schools. The first FGD (HBC FGD) comprised HBCs from Bushbuckridge who were selected from 3 home-based carer organizations. We selected home-based carers as representatives of the community because in the South African context the carers are members of the communities who either volunteer or are paid a small stipend to perform basic care and support services within the home environment. The 3 specific organizations were convenience samples based on proximity to facilitate transport to a central location. Only HBCs who could speak English were selected but most HBCs who work in Bushbuckridge do speak some English. Seventeen people were invited and the final group comprised 13 participants including 11 females and 2 males. For the second FGD (Prov/Distr FGD) 12 participants were originally invited and final group comprised of 7 (6 females and 1 male), 3 were provincial-level policy-makers, 3 district-level decision-makers and one public health specialist located in Bushbuckridge. Participants were selected using purposive sampling to ensure diversity across directorates within the Department of Health (DOH). For the third FGD (National FGD) 11 people were invited and the final group comprised 8 senior national-level policy-makers selected using purposive sampling to ensure a broad representation from different directorates within the National DOH. There were 2 females and 6 males.

We provided a board with a blank wheel that comprised coloured slices, and participants were given sticky notes to write down 2 major health topics/issues. Each participant had an opportunity to present their topics/issues and add them to the board. Health topics/issues that overlapped were grouped together and given an overarching title (eg, Access) by agreement amongst the participants. Additional topics/issues identified in step 1 (desktop review) that were not mentioned by the group, were described by the facilitator and the group decided whether they wanted them included. Following this, another round was conducted where participants wrote solutions (interventions) to address the topics/issues using sticky notes in a similar manner (Supplementary file 2). The approach fostered strong engagement and allowed the research team to refine the health topics/issues and related interventions identified in step 1. The FGDs were recorded, transcribed and analyzed qualitatively to identify topics/issues and interventions (solutions).

####  3. Modified Delphi

 During the FGDs participants were asked to vote on each health topic/issue. Topics/issues that received the highest number of votes were included in a follow up ranking process using email to each individual participant from HBC FGD and Prov/Distr FGD in order to reconcile the differences between the FGDs and to refine the list of topics/issues for the Bushbuckridge context. Three out of 6 participants from the Pov/Distr FGD completed the follow up ranking while 6 out of 13 participants responded from the HBC FGD.

We used the Borda count method to determine the overall ranking score.^41^ The Borda count method is considered a plausible approach in aggregating individual ranked preferences. The ranking was 1-13; we counted how many times each topic/issue was ranked 1-13 by all the participants. We multiplied this number by the ranking number (1-13) and then added this up to determine the total Borda count. The total Borda counts closest to zero were the ones that were ranked the highest. We initially selected the top 10 health topics/issues but some were combined and/or dropped based on being too broad or already featuring within other identified topics/issues. The top 7 health topics/issues were selected for the final CHAT board. The specific interventions under each of the topic/issues were refined and finalized by referring back to the qualitative data from the FGD and the desktop review.

####  4. Costing and Allocation of Sticker Amounts

 A total number of 70 interventions across the 7 health topics/issues were costed. Costing included both program and patient-related costs. Actuarial costs were estimated by factoring in likely utilization rates.

 For population estimates and epidemiological parameters we searched the literature from the Agincourt Research Unit. Ad hoc searches were also undertaken in PubMed, Embase, and Science direct for relevant literature on population health in Bushbuckridge. The literature were collated and assessed for relevant information that could be used to populate our costing template.

 Prices/unit costs of the components were collected from a variety of sources. Where cost information for the public sector was not available, we relied on the latest legal tariff document for the public sector. Public sector costs were assumed to be 70% of private sector costs. Medication costs were extracted from the South African National Health Laboratory Service Report 2018. For Bushbuckridge-specific parameters we relied on district level policy documents. Cost components and unit costs were developed based on researching existing programs that offered similar interventions. For information on costs of education and information provision we relied on expert consultations with stakeholders at the National Department of Health and with non-governmental organizations who have extensive experience in public awareness campaigns.

An Excel sheet was developed in Microsoft Excel to aggregate cost components. Interventions were expressed as a percentage of total costs and were converted to a relative number of stickers. Our starting point was 0.5% = 1 sticker using an existing actuarial model developed by Milliman (https://us.milliman.com/en), an international actuarial company that has experience in the adaptation of CHAT, but some allocations were revised based on the judgement of the authors. This was reasonable because the costing exercise already relied on some assumptions due to lack of data and it was important that the final sticker value represented the relative costs of interventions as accurately as possible. Where expert opinion was needed in order to verify the relative costs we reached out to individuals who were familiar with these costs. The stickers represented the monetary resources that would be required when specific interventions were selected. An overview of key data categories and sources are shown in Supplementary file 3.

Interventions were grouped together and categorized using the common classification of level of care for health interventions used in SA: Health promotion, prevention, diagnosis (screening), treatment, rehabilitation, and palliative care.^30,40^ Where categories overlapped they were merged (eg, prevention and screening).

####  5. Development of final CHAT SA Board and Supporting Materials

**Figure 1:**
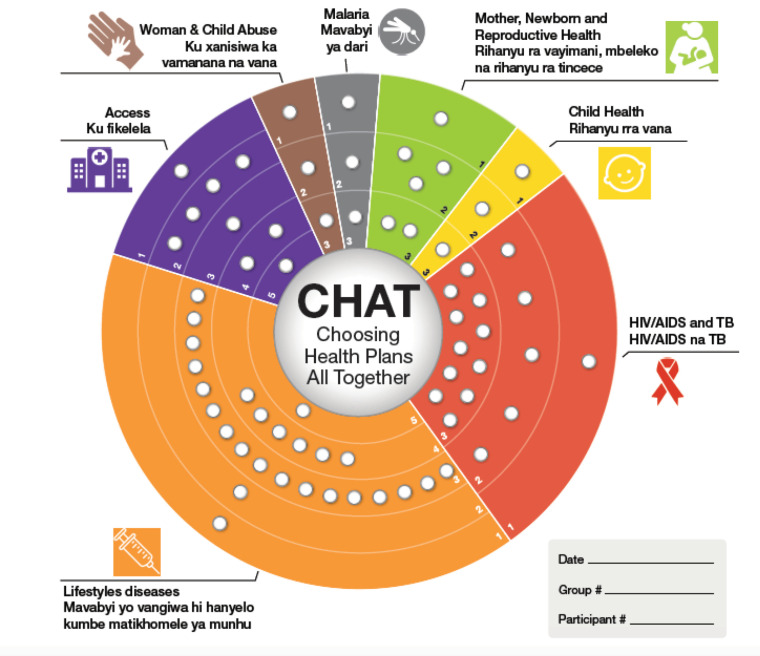
Final CHAT SA board. Abbreviations: CHAT SA, Choosing All Together South Africa; TB, tuberculosis.

The final CHAT SA board was developed based on the results from the steps above and was translated into the most widely spoken vernacular language in the study area (Shangaan). The icons and design elements were developed in conjunction with a medical artist at the National Institute of Health in the United States (Figure 1).

 A user manual was also developed which explained each category of intervention in detail by listing the specific interventions as well as context specific scenario cards to demonstrate the consequences of choices made during the CHAT exercise. These cards were developed drawing on the qualitative data that emerged from the FGD with the home-based carers to ensure the scenarios were appropriate for and relevant to the context. The materials were translated into the local language of Shangan and were checked by 2 individuals familiar with this language.

 CHAT SA was tested with a group of 11 community members (9 females and 2 males) from Bushbuckridge. This test phase was carried out in order to observe how participants interacted with the tool and the supporting materials and to ensure the tool allowed for meaningful rationing considerations. Materials were adjusted slightly and finalized post the test.

###  Data Analysis 

 Data were analyzed using thematic content analysis for the desktop review and qualitative analysis for the FGDs. Initial themes were developed by identifying the topics/issues that emerged as priorities from the Budget Speech and NHI White Paper. Following this, the policy documents were reviewed to identify themes (topics/issues) that related to those from Budget Speech or NHI White Paper as well as any that were specific to Bushbuckridge municipality or Mpumalanga province. Codes were reviewed by all authors. The FGDs were recorded, translated and transcribed in English. Data were analyzed qualitatively using thematic coding and codes were reviewed by all authors. For the FGDs we first identified themes that corresponded with those from the desktop review then identified any new topics/issues that emerged as separate themes. Sub-themes were developed to identify specific solutions/interventions under each theme and were classified according to promotion (education); prevention; diagnosis (screening); treatment; rehabilitation; palliative care.

## Results

###  Desktop Review 

 The desktop review initially highlighted 12 broad health topics/issues. These were maternal and reproductive health; neonatal and child health; school health; mental health; disability and rehabilitation services; elderly health; HIV/AIDS and TB; non-communicable diseases (NCDs); palliative services; emergency services; violence and injury; and adolescent and youth health.

###  Focus Group Discussions 

 Some common themes emerged across all FGDs, some of which overlapped with the topics/issues identified from the desktop review. These included maternal, neonatal and reproductive health (including teenage pregnancy); HIV/AIDS and TB; and NCDs including diabetes, hypertension and cancer.

 The home-based carers spent a considerable amount of time speaking about HIV related issues. They perceived that there were an increasing number of people living with HIV, with HIV positive babies still being born. They noted as reasons for this non-adherence to treatment, the lack of condom use, and a fear of disclosing HIV status to partners, family and healthcare providers.

 One of the participants said:

“*The big problem that we have is that HIV is too high. Men don’t want to go to the clinic for blood tests and they don’t want to use those condoms” *(HBC FGD, P3).

 Another participant expressed that:

“*We have many people who are defaulting nowadays…by the time they will go to the clinic, you will find that their body soldiers are down. They don’t want to take the treatment in a proper way and after they die”* (HBC FGD, P5).

 The district/provincial experts also highlighted HIV/AIDS as an issue and in particular adherence to treatment, as expressed by one participant:

“*We have very big problem with the issue of adherence on treatment…most of them are given the instructions on how to adhere to the treatment, most of them don’t adhere to treatment completely”* (Prov/Distr FGD, P7).

 The national policy-makers considered HIV and TB an issue too, especially among young women as expressed by one of the participants:

“*We don’t seem to be winning in reducing HIV infections in young women despite our many interventions to try and prevent those infections. And they have a major impact on the health of the women and the children of the country”* (National FGD, P9).

 Some specific solutions (interventions) that were mentioned under the theme of HIV/AIDS included education at the community level, making testing services youth friendly and enhancing monitoring of treatment adherence among others:

“*We should strengthen our youth health services [to address HIV and teenage pregnancy]” *(HBC FG, P5).

“*For non-adherence, we need systems… Better systems which will help the monitoring”* (Prov/Distr FGD, P4).

 Maternal and neonatal mortality was discussed as another big issue alongside teenage pregnancy across all groups. There was an overwhelming concern that sex education was not effective for young people. One of the home-based carers said:

“*Teenage pregnancy [is an issue]. You find that young woman are falling pregnant. Young people don’t listen to sex education…We don’t understand why and where the problem is” *(HBC FGD, P12).

 The lack of provision of contraceptives at schools was also a concern:

“*We have school health services but then the policy says you cannot give it [contraceptives]... to me it is like raising the demand and not supplying” *(Prov/Distr FGD, P5).

 Amongst the national experts there was agreement that maternal and neonatal mortality were issues and that teenage pregnancy was an issue in and of itself. These participants also viewed child health as related to maternal health. One of the experts said:

“*High maternal mortality rates [is an issue], and kids without moms are more likely not to survive and certainly not to thrive. So, there is an implication not only for the mom, but the entire family” *(National FGD, P7).

 Specific solutions (interventions) related to this theme included community dialogues around education, strengthening of youth health services and provision of contraceptives at schools:

“*If the schools policies can be changed that whenever we visit the school with the school health nurses, it is better to show them all the contraceptives so that they can be aware”* (Prov/Distr FGD, P7).

 A further issue identified in all 3 FGDs was NCDs and that prevention was important but not as effective as it should be. One of the provincial/district experts said:

“*I think the growing burden of NCDs is something that really is a big issue for all of us”* (Prov/Distr FGD, P3).

 One of the home-based carers expressed concerns specifically about the high prevalence of diabetes and that by the time individuals are diagnosed it is often too late.

“*Nowadays many people are dying of sugar diabetes. The problem is that this illness is hiding and by the time they find it, it is too late”* (HBC, FGD, P4).

 Similarly, one of the national experts said:

“*[There is] an explosion of NCDs, and so it is impacting on health services in a big way and if you don’t pick them up early or prevent them then we really do fear that it is going to overwhelm the health service” *(National FGD, P2).

 Solutions for NCDs that were mentioned included mobile messaging, better education at community level, vegetable gardens and others:

“*We used to have person who was responsible to teach people on how to do gardening… [this] would be a good idea [for NCDs]” *(HBC FGD, P3).

 Across all groups there was a general understanding of social determinants and the impact on health. The home-based carers spent some of the time speaking about issues of poverty specifically and how this impacts health in many different ways:

“*There are people who are still suffering [from poverty]. Our youth are not working... They are relying on child grants. The other thing that they are doing is selling themselves to men... If you ask them why are they doing this they will tell you that they want money to help their children. In that way they forgot about the illnesses that we have nowadays”* (HBC FGD, P5).

 This was similar to what some participants among the provincial/district experts thought:

“*I was looking at the social determination of health and what are the problems we are facing as the community… when you are unemployed, the issue of poverty comes in, [that is linked to] the issue of the disease of lifestyle…because when you are poor…you eat whatever comes your way without choosing and [it impacts] HIV… if you are poor being a woman, it is difficult for you to negotiate for condom usage” *(Prov/Distr FGD, P2).

 In terms of solutions for social determinants, while the HBCs did discuss some interventions to address poverty specifically, the Prov/Distr experts noted that this topic was much broader and not within the remit of the DOH:

“*That [social determinants] is a much broader area…It is actually at a different level”* (Prov/Distr FGD, P1).

“*It is not our competence”* (Prov/Distr FGD, P1).

 One issue that was particularly important to the home-based carers and the provincial/district level experts and was discussed at length was access to services, which included distance from clinics, transport issues, long queues, staff attitudes, and shortage of nurses. A considerable amount of time was spent discussing these issues during both FGDs.

 One of the home-based carers expressed that:

“…*In our clinics there is a shortage of staff. You can go there early in the morning but you can go home without getting a service. You can find that there is only one nurse who is servicing everything. [There is also] shortage of health facilities [including] mobile clinics. Some of us live far from our clinics. If we want to go to the clinics we have to get transport and we don’t have money” *(HBC FGD, P5).

 Although there has been some recognition by the government of access issues and some solutions have been created including mobile clinics, the participants highlighted that this type of intervention is now facing its own implementation and, in turn, access issues.

 Another home-based carer said:

“*You will queue for a long time. Many people are dying in the queue at the clinics and hospitals. Others are giving birth on the bench while in the queue, she will be attended by the time they see the baby coming out…The government must build health centres so that they operate day and night. This can reduce the total number of those who are collecting treatment and queueing for the whole day”* (HBC FGD, P7).

 The provincial/district experts also viewed access as an issue. Their sentiment was that number of facilities was not the problem but rather skilled personnel within the facilities:

“*We do have facilities, however access is not there in terms of number of facilities that have the skill”* (Prov/Distr FGD, P5).

“*The shortage of human resources is a real challenge”* (Prov/Distr FGD, P6).

 Related to this issue of shortage of nurses was a concern about staff attitude, especially as it related to patients accessing HIV/TB medication and young girls accessing contraceptives:

“*You can find someone who went there to collect his or her TB treatment complaining about nurses who are working slowly and again they are approaching people in a bad way” *(HBC FGD, P7).

“*The nurses must stop judging people by their age. If a girl feels ready [for contraceptives], they must not judge her” *(HBC FGD, P3).

 However, some of the provincial/district experts thought that the nurses are unfairly blamed for some of the issues and attributed their negative attitudes to poor working conditions:

“*We are short staffed…and that contributes to the negative attitude from our staff members to the community. And the reason [they might have] negative attitudes might be attributed to our staff members being burnt out…Nurses are always on the firing line because of [lack of] equipment in our facilities”* (Prov/Distr, P1).

 Solutions offered by participants to the access issues included increasing staff, improving attitudes, monitoring and evaluation at clinic level, longer operational hours for clinics and chronic medication dispensed at community centres:

“*Treatment must be there [at the community centres ] so that we don’t wait for a long time. It should come in advance so that we collect it in time. This should lower the risks of defaulting” *(HBC FGD, P11).

The FGDs demonstrated an overlap in topics across different groups (Table 1). There was stronger overlap between HBC FGD and Prov/Distr FGD than with National FGD. Some areas that were not identified by participants in National FGD were lack of information/education, elderly health, defaulting/non-adherence, and in particular access, which included distance from clinics, transport issues, long queues, staff attitudes, and shortage of nurses. Access seemed particularly important to local participants from the rural site. Mental health was raised by participants in Prov/Distr FGD and National FGD but not in HBC FGD. Topics that emerged from National FGD but not from the other 2 were disabilities, and human resources under health system. Although neonatal mortality was prioritised as a standalone issue in National FGD, it was also mentioned during the discussion under maternal mortality in the other 2 groups. Priority areas unique to Prov/Distr FGD were quality measures. Finally malaria and rape/abuse were prioritised in HBC FGD but not in the other 2 as a standalone issues.

**Table 1:** Topics/Issues Identified in the FGDs

**Topic/Issue **	**HBC FGD **	**Prov/Distr FGD**	**National FGD**
Maternal mortality/maternal and reproductive health	x	x	x
Neonatal mortality			x
Child health, including under 5 stunting		x	x
HIV/AIDS and TB	x	x	x
Social determinants	x	x	x
Lack of information/education	x	x	
Malaria	x		
Access to health services	x	x	
Rape and domestic abuse	x		
Non-compliance with chronic medication	x	x	
NCDs	x	x	x
Health system (human resource strategy, integration, material resources)		x	x
Mental health		x	x
Disabilities			x
Quality measures		x	
Elderly health	x	x	

Abbreviations: FGD, focus group discussion; HBC, home-based care; TB, tuberculosis; NCDs, non-communicable diseases.

 For some topics/issues solutions were not written down but were discussed and the desktop review supplemented discussion with specific interventions that could address the topics/issues.

###  Modified Delphi 

The differences between the 3 FGDs were reconciled using our modified Delphi. Some topics/issues were not included in the follow up ranking that was used for the modified Delpi process as they were either too broad or fell outside of the remit of the DOH. Social determinants, which included issues such as poverty, unemployment, housing and sanitation, fell into this category, even though it was important to participants. During the FGD the experts had also pointed out that the issues falling under social determinants were outside of their scope. Other topics/issues that were not included as a standalone issues were the health system (including human resource strategy, material resources, integration) and quality measures ones. These were very broad and some also featured within the other specific topics/issues. Table 2 shows the outcome from the follow up ranking using the Borda count. We initially selected the top 10 health topics/issues. We then combined newborn health with maternal and reproductive health. The monitoring and evaluation topic as well as the defaulting one did not make sense to maintain as standalone topics/issues because the specific interventions within the other topics/issues incorporated much of this so they were dropped as their own topics/issues but were maintained as part of the detail, and incorporated in the costing, in many of the other interventions. The top 7 topics/issues that remained were: maternal, reproductive and newborn health; Child Health; HIV/AIDS and TB; Lifestyle diseases; Access; Women and child abuse; Malaria. The topics/issues that were not included in the final CHAT SA board were elderly health, mental health and disabilities as these topics/issues received the lowest scores across all participants in the follow up ranking.

**Table 2:** Health Topics/Issues and Total Borda Count From Follow up Ranking With Zero Depicting the Highest Possible Score

**Health Topic/Issue**	**Borda Count**
Maternal and reproductive health (includes teenage pregnancy and adolescent health)	44
Malaria	79
Access (Improving staff attitudes, especially for family planning; Clinics open for longer hours; Increasing mobile clinics; Making chronic medicines available in communities)	34
Violence and Injury (includes rape and abuse of women and children)	92
Lifestyle diseases (sugar diabetes, cancer, hypertension)	75
Defaulting/non-adherence	68
Elderly health	109
HIV/AIDS and TB	61
Mental health	124
Monitoring and evaluation at the clinic and hospital level (includes monitoring how staff are performing)	82
Newborn health	90
Child health (includes stunting in children under 5 years of age)	105
Disabilities	133

Abbreviation: TB, tuberculosis.

###  Costing and Allocation of Sticker Value 

Table 3 shows the 7 health topics/issues, the final specific interventions and the associated costs and final sticker values. Interventions specific to each of the topics were categorized by education; prevention (and screening); and treatment. Lifestyle diseases received a fourth category – palliative care, and woman and child abuse got management instead of prevention/screening. Five unique categories were allocated to access. The intervention categories were treated as independent in implementation of the tool. Participants would be able to select some categories within a topic/issue without selecting others.

**Table 3:** Final Health Topics/Issues, Intervention Categories, Total Cost and Number of Stickers Allocated

**Health Topic/Issue and Specific Interventions**	**Total Cost (ZAR** ^a^ **)**	**Number of Stickers** ^b^
Maternal, reproductive and newborn health		
1: Education and information	4 535 704	1
2: Prevention and screening	81 815 414	3
3: Treatment	20 799 210	2
Child (<5 years ) health		
1: Education and information	4 249 151	1
2: Prevention	13 535 105	1
3: Treatment	1 162 395	1
HIV and TB		
1: Education and information	3 466 570	1
2: Prevention and screening	164 039 366	5
3: Treatment (including adherence support)	323 954 107	11
Lifestyle diseases (diabetes, hypertension, cancer)		
1: Education and information	8 721 868	1
2: Prevention and screening	3 589 976	1
3: Chronic medication and adherence support	1 017 906 916	17
4: Treatment for complications and rehabilitation	62 974 407	6
5: Palliative care	5 829 201	1
Access		
1: Improve staff attitudes (especially around family planning services for adolescents) and improve management and M&E in clinics	22 535 756	1
2: Make clinics operational for longer hours	116 454 896	4
3: Increase mobile clinics	12 076 740	1
4: Chronic Medicines (antiretrovirals, diabetes medication, hypertension meds) available at community health centres to improve adherence	74 005 752	2
5: Increase number of nurses in clinics and more pharmacists in clinics to dispense meds so wait time is shorter	29 254 008	1
Women and child abuse		
1: Education and information	2 376 035	1
2: Management of rape and abuse	798 874	1
3: Treatment	10 668 731	1
Malaria		
1: Education and information	104 274	1
2: Prevention and screening	39 850	1
3: Treatment	17 765	1

Abbreviation: TB, tuberculosis. ^a^ ZAR = 0.058 USD. ^b^ Starting point was 0.5% of total cost = 1 sticker but was revised based on professional judgement to ensure intervention values were accurate relative to one another.

###  Finalisation of CHAT SA Board and Supporting Materials

The final bilingual CHAT SA board derived from the process described here is shown in Figure 1. Each pie slice on the board reflects a topic/issue and was divided according to the different categories of interventions. The categories and specific interventions for each category were explained in detail in a user manual to accompany the CHAT SA board. The detail provided in the user manual made it clear that interventions did not overlap with one another.

**Figure 2:**
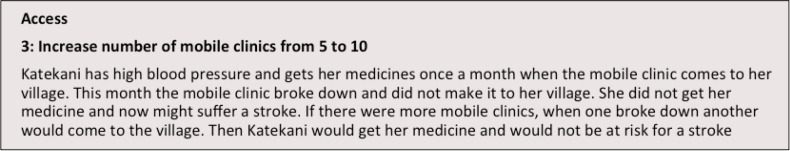
Scenario Card Under Access.

Other materials that accompanied the CHAT SA board were the scenario cards developed for each category of interventions. Figure 2 shows an example (in English) of a scenario card under “Access.”

 Following the test phase some adjustments were made. Most of these related to clarifying content in the user manual and the scenario cards to enhance understanding. Another modification was the sticker value of treatment for lifestyle diseases. This was because this category initially required too many stickers relative to the other categories and if not chosen allowed participants to select almost all the other interventions on the board, obviating the need for priority setting once this was excluded. Due to the costing relying on high degrees of professional judgment, this adjustment was reasonable.

## Discussion

 The modification process of the CHAT tool for a rural South African context identified 7 health topics/issues and related interventions. As the country moves towards UHC with limited resources, tough decisions will need to be made at the national and provincial level about which interventions will be covered, and at the local level, about how services will be delivered. Some of these options are included in the final CHAT SA board, which reflects both priority options of policy-makers/experts and of community members, and demonstrates some of the context specific coverage decisions that will need to be made. The CHAT SA is a context-specific tool which can be used for deliberative engagement and is relevant and appropriate for the rural context in SA. Because of the specificity of the modified tool for a rural context the acceptability of the tool will likely be high among rural community representatives. This in turn could impact the potential success of its implementation. If implementation of the tool is successful in Bushbuckridge it has the potential to generate more meaningful public engagement, and may be useful in eliciting social views around these health topics/issues by identifying priorities of the local community. This in turn has the potential to inform decision-making at the different levels with regard to a health service package.

The method used to modify the CHAT tool for a rural SA context included a high degree of community engagement and was described in detail. Most previous modifications of CHAT have followed a top down approach whereby the topics/issues for inclusion in the CHAT board are determined by experts with no or minimal community engagement.^20,21,23^ Some previous CHAT work has included initial preparatory discussions with experts and patient representatives to identify key issues/questions to include in the various versions of CHAT.^25,27^ The iterative participatory approach that we followed involved a high degree of consultation with experts/policy-makers and community members over time, and drew on lessons from the CHAT exercise itself to engage with the different stakeholders.

The participative process we followed allowed us to identify health topics/issues and related interventions specific for Bushbuckridge community to be included in the CHAT SA tool. The difference in topics/issues amongst the groups, specifically between national policy-makers and the community home-based carers demonstrates that community engagement is important in not only identifying social values for final coverage options but also for identifying the initial topics/issues and related interventions that should be weighed up when thinking about potential health benefits packages. This is different from the common approach to priority setting which relies on the views of expert decision-makers in defining the agenda even in countries where participatory governance structures are in place. Public engagement in decision-making where it exists has conventionally been implemented once the potential options have been predefined by the experts, even if at times influenced by a group of the public who has secured a voice, and with little consideration of the appropriateness of the method used.^10,42^ This paper demonstrates that if an initial inclusive and consultative approach is not followed issues like access, for example, may not be comprehensively considered ahead of time and thus may be absent from the package of potential coverage options. Issues like access, however, do not simply impact implementation but ought to be taken into account when defining health service packages and allocating the budget. The agenda, ultimately, may benefit from being set not simply by decision-makers, and those that shout the loudest, but also by the broader public and the modification process we followed may be useful in this regard.

 SA is committed to public engagement in decision-making but existing structures either do not exist or do not operate as intended. This may be due, in part, to the absence of appropriate context-specific tools that facilitate public engagement in decision-making around resource allocation. CHAT SA could fill this gap by strengthening existing structures like the community health committees where implementation of the tool might be helpful in ensuring the participation of the committees in the governance of and priority setting process for local clinics. CHAT SA could also be useful in initiating the establishment of new national structures that make use of this tool, to ensure that the public’s voice is included in decision-making.

Although lessons from the modification of CHAT may be applied nationally, the tool itself was modified for a specific rural community and may not be appropriate for implementation nationally. SA is not homogenous with different health outcomes and challenges across its 9 provinces. The CHAT SA tool while useful for a rural context may need to be adjusted for further implementation, especially in urban areas. This will require additional research locally. Herein lies the dilemma of how to develop a public engagement tool for priority setting in health that is specific enough for a local context but pragmatic enough to be applied across the country in different settings, however CHAT SA may also offer some answers. The inclusion of national policy documents and national policy-makers in our study ensured that many of the national priorities featured in the modification of the CHAT tool, although these were refined for the specific rural context. The adjustment of the CHAT SA tool for different contexts in SA may be necessary in order to elicit broader social values but the existing modified tool can serve as a basis for this. In future potential modification processes of CHAT (for health service packages) for different South African contexts the initial steps which consisted of the desktop review of national policy documents as well as the FGD with national policy-makers need not be repeated. However, it would be necessary to replicate the community engagement component in order to identify specific topics/issues and related solutions/interventions to include in the CHAT board for the different South African contexts, as well as to ensure translation of the CHAT board and supporting materials in the local vernacular language(s). It is likely, however, that many of the topics/issues will remain because despite its heterogeneity the top causes of death are similar across provinces^43^ but some service delivery challenges may differ across local contexts which ultimately impacts specific health interventions. The tool would also need to be updated every few years in the different settings in order to ensure it remains relevant as the health terrain changes.

 Our modification process demonstrates that local terms that are acceptable to the community may be more important than scientific ones. We initially tested the term NCDs with the HBCs to capture the issues of cancer, type 2 diabetes (“sugar diabetes”) and hypertension but the term lifestyle diseases was deemed more appropriate and acceptable. Although lifestyle diseases may have some negative connotations the community perceived it to be easily understandable. Subsequent modifications might reveal other more appropriate terms for similar categories.

 A further lesson is that our modification process was resource and time intensive, and took a year to complete, and therefore may not be replicable when an urgent coverage decision is required about a specific health intervention to include in or remove from the health service package. In order to avoid this dilemma, decision-makers should be encouraged to support the development of context specific engagement tools that should be used on a regular basis within the priority setting entities or institutions that evolve in SA. These engagement tools should be applied in determining the health service packages and in subsequent decisions about new interventions.

###  Limitations

 A limitation of our study was that because cost data were not available for every intervention the costing component included a number of assumptions and a great level of professional judgment in developing our allocations and sticker values. We aimed to ensure the relative costs of interventions were as accurate as possible relying on expert opinion where necessary. Future modifications of CHAT for SA would likely need to do the same, however, as more cost effective analyses of interventions become available due to SA’s commitment to evidence-based priority setting, more reliable costing data will be available for some interventions and CHAT modifications will be able to make use of these data. Other countries that have better costing data would not need to rely on as many assumptions and professional judgement but in settings with similar data limitations it would be important to consult with people who have experience of program costs.

 Related to this, the costing of the interventions did not fully account for delivery of these interventions at high quality. Our costing model incorporated additional training, supervision and management support for interventions delivered through the health system in order to improve quality but comprehensive quality improvements would require addressing many of the health system constraints (beyond access issues), and was not feasible for the scope of this project.

 Another limitation related to professional judgement was that this applied to not only costing data but also in refining some of the interventions that were ultimately included in the final CHAT board. The research team works closely with health policy-makers and the familiarity may have influenced some decisions inadvertently. The decision to exclude social determinants of health as a topic seemed reasonable in light of policy-makers within the DOH viewing these issues as outside their remit. A CHAT tool for SA that incorporates these social determinants of health and that informs multisectoral collaboration in priority setting could be useful in the future.

 A further limitation was that the participants were skewed in terms of gender- while the national FGD featured mostly males, the majority of participants in the HBC FGD and the Prov/Distr FGD were females. If more males had been included some of the topics/issues may have differed.

 A final limitation is the generalizability of the final CHAT tool. The provincial/district experts represented a range of managers from different directorates who are responsible for dealing with a variety of health issues from planning to implementation challenges and the home-based carers are embedded within the community at the forefront of daily health issues. The inclusion of these participants in the FGDs ensured that context specific topics/issues were identified. However, other topics/issues may have emerged, or could have been ranked higher if additional participants had been included in the FGDs. This would have impacted the final topics/issues that were included in the CHAT board.

## Conclusion

 This research adds to the body of work on public engagement for priority setting in health and provides practical lessons for the modification of deliberative engagement tools like CHAT. This is especially relevant as countries move towards UHC and engagement methods are needed to ensure the public is included in the priority setting process. Methodologies that include participatory principles and that involve the public in setting the agenda, are useful and feasible for the modification of engagement tools like CHAT and can be applied in different country contexts in order to ensure these tools are relevant, appropriate and acceptable. In order to overcome some challenges in the modification process that generates a highly context specific engagement tool the inclusion of national policy documents and national experts should be considered. This would facilitate a pragmatic approach to the application of the tool in different settings within countries. This participatory modification approach could result in engagement tools that may, through their implementation, ultimately ensure better priority setting approaches that are not only evidence-based but also ethical, legitimate, sustainable and inclusive.

## Acknowledgements

 This research was supported by The South African Medical Research Council (SAMRC-RFA-EMU-02-2018) and the International Decision Support Initiative.

## Ethical issues

 Ethical clearance was obtained from the Human Research Ethics Committee (Medical) of the University of the Witwatersrand, Johannesburg, South Africa [Clearance certificate number M161009]. Free and informed consent from all participants was obtained.

## Competing interests

MD and her institution may benefit from paid licenses for the use of the electronic version of CHAT^©^. This study did not develop an electronic version but there may be the possibility for this in implementation of the tool.

## Authors’ contributions

 AT, KH, MD, NC, and KK contributed to the conception and design of the study; data acquisition and interpretation was conducted by AT and AE; drafting of the manuscript was completed by AT; revision of the manuscript was done by all authors; administrative, technical/material support was provided by AK, MD, AE, and NC; funding support was secured by AT and KH.

## Authors’ affiliations

^1^SAMRC/Wits Centre for Health Economics and Decision Science - PRICELESS, School of Public Health, Faculty of Health Sciences, University of Witwatersrand, Johannesburg, South Africa. ^2^Department of Bioethics, National Institutes of Health, Bethesda, MD, USA. ^3^School of Public Health, Faculty of Health Sciences, University of the Witwatersrand, Johannesburg, South Africa. ^4^MRC/Wits Rural Public Health and Health Transitions Research Unit - Agincourt, School of Public Health, Faculty of Health Sciences, University of the Witwatersrand, Johannesburg, South Africa. ^5^New York Academy of Medicine, New York City, NY, USA.

## Endnotes


[1] CHAT has a licensing fee that is waived where its use/modification is in collaboration with one of the developers.


## Supplementary files


Supplementary file 1. Documents Included in the Desktop Review.

Supplementary file 2. FGD Guide.

Supplementary file 3. Data Categories and Sources.
