# Supplementary material for: CHAT SA: Modification of a Public Engagement Tool for Priority Setting for a South African Rural Context
Source: Int J Health Policy Manag. 2020 Jul 8;11(2):197–209. doi: 10.34172/ijhpm.2020.110 (PMC9278606; doi:10.34172/ijhpm.2020.110)
Supplement: Supplementary file 1 — Documents Included in the Desktop Review. [file ijhpm-11-197-s001.pdf]

Supplementary file 1. Documents Included in the Desktop Review

**National policy documents**
NHI White paper 2015
Budget speech 2017, transcript
Negotiated Service Delivery Agreement, 2010-2014
National Development Plan 2013-2030
The Primary Healthcare Package for SA, 2000
Department of Health Strategic Plan 2014-2019
Maternal, Newborn, Child and Women's Health and Nutrition Strategic Plan 2012-2016
South Africa's National Strategic Plan for a Campaign on Accelerated Reduction in Maternal Mortality in Africa (CARMMA), 2012
Adolescent and Youth Health Policy 2016-2020
National Plan of Action on Ageing 2006-2016
National Mental Health Policy Framework and Strategic Plan 2013-2020
Strategic Plan for the Prevention and Control of Non-Communicable Diseases 2013-17
Framework for Disability and Rehabilitation Services 2015-2020
**Provincial policy documents**
Mpumalanga Annual Performance Plan 2016/2017
**District policy documents**
Ehlanzeni District Health Plan 2015
Bushbuckridge local municipality Integrated Development Plan 2011-2016
