# Supplementary material for: CHAT SA: Modification of a Public Engagement Tool for Priority Setting for a South African Rural Context
Source: Int J Health Policy Manag. 2020 Jul 8;11(2):197–209. doi: 10.34172/ijhpm.2020.110 (PMC9278606; doi:10.34172/ijhpm.2020.110)
Supplement: Supplementary file 2 — FGD Guide. [file ijhpm-11-197-s002.pdf]

Supplementary file 2. FGD Guide

Explain the purpose of the focus group

Round 1:

Show the board with an empty wheel with different color segments, hand out 3 sticky notes to each participant and explain that each participant will have a turn to add the 3 sticky notes with a health topic/issue to the wheel.

1) Which 3 health issues/ topics can you think of that are important for
    - Bushbuckridge community (for HBC FGD)?
    - Mpumalanga Province/ Bushbuckridge sub-district (for Prov/Distr FGD)?
    - The Country (for National FGD)?

– list each one on a sticky note

Give the participants 15 minutes to think about the issues and to write them down then go around the room giving each participant a chance to add their sticky notes to the wheel and describe the 3 issues/ topics

Probing questions:

- Why do you think this issue is important?
- Can you tell us more about this health issue and who it is impacting?
- Is there anything more you would like to add about this issue?
- If the issue is not clear ask follow up questions for clarity?

2) Do others agree that this is an important issue? (Give participants a chance to reflect on what has been said).

Tell the participants to look at the wheel with all the sticky notes and explain that you will group the sticky notes together where issues overlap or are connected, and you will place them in one segment of the wheel. Ask the following:

3) Does everyone agree that these sticky notes overlap or can be grouped together? (If no, ask why and give participants an opportunity to reposition the sticky notes).

Once there is agreement about the grouping, explain to the participants that you will now categorise the issues under an overarching term. Suggest terms that have emerged from the discussion and ask:

4) Does everyone agree with this categorization for these common issues? If there is disagreement, ask participants to give justification for this and to propose a more appropriate category term.

Once the categorization has been finalised ask the participants to vote for each of the categories:

5) I will call out each category one by one, please raise your hand if you feel that this category is important and should be kept on the board (note number of votes for each, all with majority vote keep on the board).

Refer to list of topics/issues from desktop review- If there are any issues that have not emerged from the discussion call them out one at a time and ask participants to raise their hands if they would like them included. If there is time, allow participants to reflect on the additional issues.

Round 2:

Explain to the participants that they will now be given more sticky notes and will be asked to write solutions on the sticky notes for each of the categories on the board. Begin with one of the categories, give participants 5 minutes to write down some solutions (some may have emerged from round 1 already), start with one of the participants and go around the table:

6) Please explain your solution to the group and stick it on the board under the category.
7) Do others agree that this is a good solution?
8) Does anyone have anything to add to this particular solution?

Once all participants have had a chance to voice solutions related to all of the categories on the board, thank participants for their participation and conclude the discussion.
