# Supplementary material for: CHAT SA: Modification of a Public Engagement Tool for Priority Setting for a South African Rural Context
Source: Int J Health Policy Manag. 2020 Jul 8;11(2):197–209. doi: 10.34172/ijhpm.2020.110 (PMC9278606; doi:10.34172/ijhpm.2020.110)
Supplement: Supplementary file 3 — Data Categories and Sources. [file ijhpm-11-197-s003.pdf]

Supplementary file 3. Data Categories and Sources

Data category	Parameter	Source
**Population estimates**	Population of all age groups<br>Estimated pregnant women	Integrated Development Plan: IDP 2011-2016
**Epidemiology parameters**	Disease burden<br>Medicine and technology need and usage<br>Hospitalization<br>Outpatient visits	Ehlanzeni District Health Plan (DHP) 2017/2018<br>Literature review
**Infrastructural and managerial parameters**	Service deliver platforms for health<br>Human resources for health for health	Ehlanzeni District Health Plan (DHP) 2017/2018
**Direct medical costs**	Medication	South African National Health Laboratory Service
	Examinations and investigations	Uniform Patient Fee Schedule 2018<br>The Lives Saved Tool
	Health workers wages	Department of Public Service and Administration
**Education provision**	Mobile phone messaging<br>Media campaigns	Stakeholder consultations
